# Omega-3 PUFA vs. NSAIDs for Preventing Cardiac Inflammation

**DOI:** 10.3389/fcvm.2018.00146

**Published:** 2018-10-23

**Authors:** Jiayu Ye, Sanjoy Ghosh

**Affiliations:** Irving K. Barber School of Arts and Sciences (IKBSAS), Department of Biology, University of University of British Columbia, Kelowna, BC, Canada

**Keywords:** inflammation, docosahexaenoic acid (DHA), EPA, aspirin, NSAIDs

## Introduction

Inflammatory cell accumulation occurs in the cardiac muscle during cardiac injury and repair (1). From an evolutionary perspective, inflammation is required for immunosurveillance and host defense. However, such cardinal signs of acute inflammation, such as redness, pain, swelling etc., due to injury or infection. Might be typically absent in chronic low-grade inflammation (LGI). Current literature suggests that chronic low-grade inflammation (LGI) is a primary causative factor behind chronic diseases like cardiovascular diseases (CVD), non-alcoholic fatty liver disease (NAFLD) and obesity (2).

## Common mechanisms for NSAIDs

NSAIDs are anti-inflammatory drugs which as a class block the generation of prostaglandins (PGs), leukotrienes (LT) or epoxides (3), which are upregulated during inflammation Therefore, NSAIDs are commonly used for prevention of multiple chronic inflammatory conditions including CVD. The major enzyme participating in PG biosynthesis is cyclooxygenase (COX), which is subdivided as constitutive COX-1 and inducible COX-2 forms. These isoforms of COX show differential activity in inflamed tissues. COX-2 is expressed 10- to 80-fold, whereas COX-1 expressed 2- to 4-fold (4). Both COX isoforms are responsible for converting arachidonic acid (ARA) to intermediate PGs, the PGG_2_, and the PGH_2_. ARA is the precursor of eicosanoids which is cleaved by phospholipase A_2_ (PLA_2_) from membrane phospholipids. Then thromboxane synthase and various isomerases are activated which generates thromboxane A_2_ (TxA2) and PGs (PGE_2_, PGF_2α_, PGD_2_, PGI_2_) (5). These four PGs have the common function of vasodilation as well as increasing permeability of membranes (thus promoting “redness” due to increased blood flow). PGE_2_ and PGF_2α_ are mainly produced by monocytes and macrophages, mast cells produce PGD_2_ and endothelial cells produce PGI_2_ (6). Long-term treatment with NSAIDs lower beneficial PGs as well (7). PGE_2_ and PGF_2α_ control water and electrolyte absorption and maintain secretion in gastric mucosa. Thus, NSAIDs can decrease the secretion of mucous-bicarbonate barrier between the gastric lumen and epithelial cells. Subsequently, in contact with low pH of the stomach, epithelial cells are killed and the integrity of the mucosa is lost, causing ulceration (7).

## Aspirin

Aspirin is a widely used anti-inflammatory drug. Aspirin inhibits the COX activity by acetylating the hydroxyl group on COX, which specific acts on serine residues. This leads to the irreversible inhibition of COX, as well as causes the ARA binding restriction (8).

Aspirin is easily and quickly absorbed in the GI tract and hydrolyzes to salicylic acid (SA) in the stomach and intestine. However, SA and aspirin can strongly bind to albumin. This avoids the hydrolysis of aspirin too fast (9), as albumin concentration often decreases dramatically under acute inflammation due to the formation of complex albumin-hyaluronic acid or due to decreased albumin synthesis (9, 10). Thus, SA and aspirin hydrolyze faster under acute inflammation. In this case, regulating the range of SA (or aspirin dose) concentration is critical in order to avoid further adverse effects. The half-life of aspirin is relatively short, for 15–20 min in adults. Aspirin inhibits the PGs production mainly due to the blockage of COX-2. However, aspirin also inhibits the cytoprotective PGs in gastric mucosa, which impairs the integrity of epithelial cells and also destabilizes the lysosomal membrane (11).

## Indomethacin

Indomethacin is a NSAID, and is effective against fever and pain. Like aspirin, indomethacin is a non-selective NSAIDs, which block both COX-1 and COX-2 (12). Thus, PGE_1_ and PGE_2_ in gastric mucosa can be reduced, resulting in gastric and intestinal ulceration (11). Similarly, with the inhibitory effect of TxA_2_, the platelet aggregation ability decreases dramatically to induce bleeding (13). Studies also show that indomethacin can potentially increase the blood pressure in patients (14). It is easily absorbed by the GI tract and bind with the protein in plasma and injured tissues, specifically albumin as aspirin.

## Ibuprofen

Ibuprofen is an effective analgesic and an antipyretic and especially recommended for children because of its better safety profile. Ibuprofen needs longer time than other NSAIDs as an antipyretic and it has a more intense effect on pain relief than aspirin (15). The advantage of this drug is the lighter side effects on the GI tract. High dose for long-term use is mainly for the chronic inflammatory diseases, including arthritis (15).

## Recent problems with using NSAIDs in cardiac diseases

NSAIDs like aspirin has been used for decades to protect against low grade inflammation in cardiovascular disorders. It was initially thought to be safe and effective against systemic inflammation affecting the cardiovascular system (16, 17). However, evidence of benefit as not been consistent (18) and is plagued by major side effects like GI bleeding, which can make this therapeutic approach questionable in vulnerable populations (19). In essence, a core impact of NSAIDs is to inhibit COX activity. However, COX activity, especially COX-2, is responsible for also maintaining aorta function. COX2 disruption can harden aorta leading to aortic fibrosis (20) and atheroclerosis (21). In addition, inhibition of COX2 reduces the benefits of statin on cardioprotection (22). This might be the reason why all NSAIDs like ibuprofen and naproxen in addition to aspirin in recent years have demonstrated cardiovascular effects including heart attacks (23, 24). More importantly, according to a recent study, long-term users of aspirin have >30% increased chance of a cardiovascular event upon withdrawal of the drug (25). Therefore, preventative therapies that avoid long term NSAID use is warranted in chronic inflammatory diseases including CVDs.

## Omega-3 PUFA

Omega-3 PUFA are polyunsaturated fatty acids with the first double bond on the third carbon from the terminal methyl end. Fish and flaxseed oils are rich in omega-3 PUFA with protective functions for the heart (26), liver (27), and brain (28). The major fatty acids contained in the fish oil supplement are eicosapentaenoic acid (EPA) and docosahexaenoic acid (DHA), the long chain members of the omega-3 family. In contrast, flaxseed oil is mainly composed of alpha-linolenic acid (ALA), the parent omega-3 PUFA.

## Mechanism of anti-inflammatory response

Similar to NSAIDs, omega-3 PUFA, especially EPA and DHA inhibit the production of pro-inflammatory eicosanoids. However, instead of blocking COX activity, they use the same COX to increase the production of anti-inflammatory eicosanoids by providing a different substrate. Twenty-carbon omega-3 PUFA and ARA compete with each other for the use of the COX enzyme. This increases the production of the anti-inflammatory mediators like LTB_5_ and PGE_3_ from EPA and at the same time limits the inflammatory LTB_4_ and PGE_2_ production from ARA (29). In this case, the mucosa protective PGs (PGE_2_) are still available, albeit reduced. As a result, the side effects caused by anti-inflammatory drugs are drastically reduced. Given the basic differences in the mechanism of NSAIDs and omega-3 PUFAs in blocking COX vs. providing an alternate substrate to COX enzyme, the timeline for actions are vastly different. NSAIDs are more acute in action due to direct enzymatic blockade, whereeas omega-3 PUFAs act slower due to its gradual replacement of membrane phospholipid ARA, which might take weeks if not months to have a biologically plausible effect. Clearly, NSAIDs are preferred for acute inflammatory challenges resulting from physical injury or trauma whereas omega-3 PUFAs are at best a long term, mild anti-inflammatory solution. Thus, consuming omega-3 supplement can be considered as a preventative therapy, alternate to NSAIDs on resolving long-term chronic inflammatory stage, with some major differences as listed in Table 1 (65).

**Table 1:** Comparison of anti-inflammatory role between omega-3 PUFA and NSAIDs.

	**Omega-3 PUFA**	**NSAIDs**
Main effect	Reducing inflammatory eicosanoids production (30)	Blocking inflammatory eicosanoids production (31)
Mechanism	Compete with ARA for COX binding sites (30)	Broadly block COXs activity (31)
Lipid profile	↓TC, ↓TG, ↓LDL, ↑HDL (32)	↑LDL, ↑ TG, ↑ TC, ↓HDL (33, 34)
Blood pressure	↓(35)	↑(36, 37)
Cytokine	↓IL-1 (38), ↓TNFα (38), ↓IL-6 (39)	↓IL-1, ↓TNFα, ↓IL-6 (40)
ROS	↓(41)	↑(42)
Low-dose effect^*^	Slowing renal dysfunction (43) Reduce seizure and improve epilepsy (44) Improvement on mild to moderate depression (45) Decreased oxidative stress in type 2 diabetes (46)	Primary Cardiovascular Diseases Prevention (47) cerebrovascular disease prevention (48) analgesic effect on dental pain(49) and osteoarthritis pain (50) inhibitory effect on tumor cell growth and metastasis (51) Intraventricular Hemorrhage prevention (52)
High-dose effect^*^	Substitution of NSAIDs in rheumatoid arthritis (53) Secondary prevention of cardiac disease (54) Slow renal dysfunction (43)	First-line desmoid tumor treatment (55) Long-term treatment attenuates cystic fibrosis (56)
Side-effect	↑ LDL, ↑ HDL, ↑ insulin resistance (57) Higher risk of sudden cardiac death (58) ↑ lipid peroxidation (59, 60)	Gastrointestinal bleeding (61) Increased risk of spinal fusion after sugery (62) Increased risk of coronary heart disease (63) Worsened kidney function (64)

* *Omega-3 low dose < 1.2 g/d, high dose >2 g/d. NSAIDs dose is various depending on the drug*.

In addition to modulating PGs, EPA and DHA produce lipid mediators responsible for anti-inflammation and resolution: resolvins and protectins (66). Due to the different substrates of resolvins' production, they are divided into E-series from EPA and D-series from DHA (66). Although they are from different sources, they show very similar effects on preventing inflammation. Both of them increase with the presence of aspirin or with higher EPA/DHA consumption, which are stimulated by the aspirin acetylated COX-2. As mentioned before, COX-2 is the rate-limiting enzyme promoting the synthesis of pro and anti-inflammatory eicosanoids, depending on the different substrates. COX-2 dependent resolvins attenuate inflammation and block the human neutrophil transendothelial migration by competing for the leukotriene B4 receptors (BLT1) with LTB4 (67). Protectins are the other group of new pro-resolving and anti-inflammatory lipid mediators, which are derived from DHA only (66). They block the immigration of T-cells, promote the T cell apoptosis, and reduce the potent inflammatory factor TNFα (68).

Other than directly affecting the eicosanoid pathway, ALA, EPA, and DHA also helps to reduce pro-inflammatory cytokines, including TNFα, IL-1, and IL-6 (30). These potent cytokines initiate the cascade of pro-inflammatory mediators, including cytokines, chemokines and adhesion molecules following injury. This leads to the high recruitment of immune cells, such as neutrophils, monocytes, B cells and T cells. In most acute and chronic inflammatory diseases, it has been shown that omega-3 PUFA attenuates the inflammatory response by reducing the inflammatory hallmarks (30). In cardiac diseases, omega-3 PUFA can inhibit the secretion of lipopolysacchrides (LPS). This limits the initiation of LPS-induced inflammatory pathway, including NF-κB and toll-like receptor 4 (TLR4) (69). Along with such inhibition of pro-inflammatory signaling, nitric oxide (NO) production increases (45). This leads to improved endothelial function (70).

Considering of the close relation between inflammation and oxidative stress, omega-3 PUFA can also lower oxidative stress through increased cellular antioxidant capacity. However, this result can only be reached with over 3.4 g/day EPA/DHA consumption (71). Having a high dose of omega-3 PUFA on the other hand can cause excess fatty acid accumulation, which potentially can also increase oxidative stress, given that omega-3 PUFAs have multiple double bonds amenable to oxidation.

## Potential risks of omega-3 PUFA supplementation

Inflammation exists to fight off infection or injury. Thus, while LGI may be perceived as detrimental, acute inflammation especially in the context of infection is a protective response that needs to be sustained at least for some time. In a chronic inflammatory state, such as rheumatoid arthritis (RA), EPA/DHA reduce RA inflammation and benefits the patient. However, the similar effects during infection or tumor surveillance can result in a negative health outcome (72–74). In 2005, IOM summarized that intake of 0.9–9.4 g/day of EPA and 0.6–6 g/day of DHA was linked to an impairment of immune responses. It is now known that DHA and EPA can both improve and impair host resistance to a number of pathogens (75, 76). These adverse outcomes of omega-3 intake were observed with bacterial, fungal and viral pathogen models (77). Given the potent anti-inflammatory effects of DHA and EPA, it is thus conceivable omega-3 PUFA can be both helpful or detrimental specific to the disease context. Moreover, like most nutrients or anti-inflammatory drugs, there is a potential for negative health effects under excess intakes.

## NSAIDs and omega-3 PUFA in combination?

An intriguing idea would be to use both low dose NSAID and long chain omega-3s like DHA/EPA in combination for prevention of cardiac and other LGI states. In theory, as both these classes of drugs act on the same COX/LOX pathway, the requirements/dosing of each might be lower due to their synergistic effects. The problem with such an approach is that long term safety of omega-3 supplementation in the pill form still remains unestablished in patients with various LGI states including CVD. With recent reports of long term ill effects of NSAIDs at the current dosing levels in cardiac patients, there is evidence that it might be risky to carry on such a trial for potential negative effects on coagulation (78).

In conclusion, the effectiveness of NSAIDs for acute inflammation has not translated to a safe strategy for long term prevention of CVD. Controversy also surrounds the long term impact of omega-3 PUFA as a preventative measure against chronic low grade inflammation (77). However, in patients unable to take NSAIDs in the long term due to GI or bleeding problems, due to the similarity in their mechanism of action, low dose omega-3 PUFA could be a substitute to prevent LGI associated with cardiovascular diseases.

## Author contributions

JY wrote the paper. SG edited and revised the paper. Both authors read and approved the final manuscript.

### Conflict of interest statement

The authors declare that the research was conducted in the absence of any commercial or financial relationships that could be construed as a potential conflict of interest.
